# *Wolbachia*, *Cardinium* and climate: an analysis of global data

**DOI:** 10.1098/rsbl.2019.0273

**Published:** 2019-08-21

**Authors:** J. Charlesworth, L. A. Weinert, E. V. Araujo, J. J. Welch

**Affiliations:** 1 Department of Genetics, University of Cambridge, Downing Street, Cambridge CB2 3EH, UK; 2 Department of Veterinary Medicine, University of Cambridge, Madingley Road, Cambridge CB3 0ES, UK

**Keywords:** endosymbiosis, biogeography, Köppen climates, beta-binomial modelling

## Abstract

Bacterial endosymbionts are very common in terrestrial arthropods, but infection levels vary widely among populations. Experiments and within-species comparisons suggest that environmental temperature might be important in explaining this variation. To investigate the importance of temperature, at broad geographical and taxonomic scales, we extended a global database of terrestrial arthropods screened for *Wolbachia* and *Cardinium*. Our final dataset contained data from more than 117 000 arthropods (over 2500 species) screened for *Wolbachia* and more than 18 000 arthropods (over 800 species) screened for *Cardinium*, including samples from 137 different countries, with mean temperatures varying from −6.5 to 29.2°C. In insects and relatives, *Cardinium* infection showed a clear and consistent tendency to increase with temperature. For *Wolbachia*, a tendency to increase with temperature in temperate climates is counteracted by reduced prevalence in the tropics, resulting in a weak negative trend overall. We discuss the implications of these results for natural and introduced symbionts in regions affected by climate change.

## 1. Introduction

Bacterial endosymbionts can exert profound effects on their hosts, for example, by manipulating host reproductive biology to maximize their own vertical transmission [1–3]. Infection with such symbionts is very widespread among arthropods, with the genera *Wolbachia* and *Cardinium* having been estimated to infect, respectively, around 50 and 12.5% of all terrestrial arthropod species [3]. Nevertheless, symbiont prevalence (i.e. the proportion of infected individuals within a population) varies widely, and the reasons for this remain unknown. Several factors may shape variation in prevalence, including costs of reproductive parasitism [4], benefits of protection against viral pathogens [5] or host dispersal patterns [6]. Another putative influence on endosymbiont prevalence is environmental temperature [7]. Laboratory studies suggest that endosymbionts are more susceptible to thermal stress than their hosts [8–10], and the physiological costs or benefits of endosymbiont carriage or transmission might be temperature-sensitive [7]. Positive temperature clines have been observed for *Cardinium* infection in *Culicoides* midges in Israel [11], for the global prevalence of *Wolbachia* infection in Lepidoptera, and across tropical/temperate gradients in Australian Diptera [12–14]. However, these are isolated results, and the prevalence of *Wolbachia* in insect populations, for example, remains remarkably constant across broad continental scales [15].

Weinert *et al.* [3] collated a database of published PCR screens for *Cardinium* and *Wolbachia* in wild populations of terrestrial athropods and developed a maximum-likelihood modelling framework that they used to test hypotheses about endosymbiont incidence in different arthropod groups [3]. Here, we greatly extended their database, and modified their methods to test whether temperature clines for prevalence of *Wolbachia* and *Cardinium* obtain more generally. *Wolbachia* is of special interest because of its use as a bio-control agent for mosquito disease vectors [5,6]. *Cardinium* provides an interesting contrast because it adopts many of the same transmission strategies as *Wolbachia*, and is also known to infect both pests [16] and disease vectors [11,17] but is present at a much lower incidence.

## 2. Results

**Figure 1.**
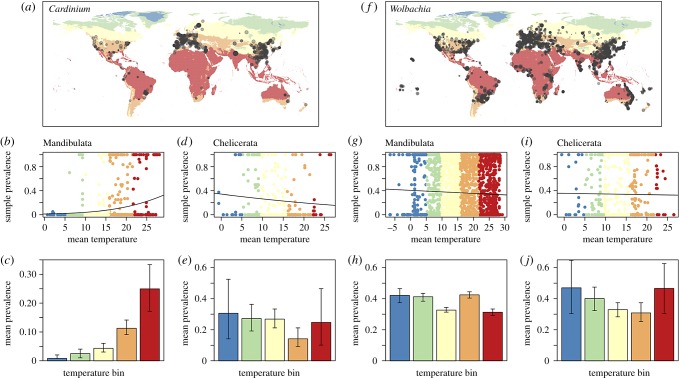
Climatic variation in the prevalence of *Cardinium* (*a*–*e*) and *Wolbachia* (*f*–*j*) infection in terrestrial arthropods. (*a*,*f*) World maps, with colours corresponding to mean temperature over the period 1970–2000. Points indicate population screens, with point size indicating the number of individuals sampled. (*b*,*d*,*g*,*i*) Regression of mean prevalence (logit transformed) on the mean temperature of sampling location; the best-fit lines correspond to the three-parameter models reported in table 1. (*c*,*e*,*h*,*j*) Illustrative plots, showing the estimated mean prevalence for populations falling within temperature bins, coloured to match the middle panels, and centred on the mean temperature for the five major Köppen zones: polar (blue); continental (green); temperate (yellow), arid (orange) and tropical (red). Confidence intervals correspond to two units of log-likelihood. Separate results are given for the two major clades of arthropods, namely Mandibulata (mostly insects, but also wingless hexapods, crustaceans and myriapods), and Chelicerata (ticks, mites, spiders and relatives). (Online version in colour.)

We extended the database of Weinert *et al.* [3] to yield 135 876 arthropods screened for *Wolbachia* and *Cardinium,* drawn from a total of 320 publications. When sampling location was specified, we obtained an estimate of mean temperature between 1970 and 2000 [18], and assigned each sample to a Köppen climate zone, which summarizes multiple ecologically relevant variables [19,20]. The data are diverse taxonomically (with hosts from more than 40 arthropod orders), and geographically (with 27/31 Köppen climates, including all higher-level zones: tropical, arid, temperate, continental and polar). The database is available as electronic supplementary material, table S1, and is summarized in figure 1 and electronic supplementary material, figures S1–S3.

We first consider *Cardinium* (figure 1*a*–*e*). This symbiont has very different prevalences in the two major arthropod groups, namely Chelicerata: mites, ticks, spiders and relatives, and Mandibulata: mainly insects, but also wingless hexapods, crustaceans and myriapods [3,21], so we consider these two groups separately.

For *Cardinium* infection in Mandibulata (figure 1*b*,*c* and table 1*a*), there is a clear trend for increasing infection with temperature (figure 1*c*). This is confirmed by a regression analysis (figure 1*b* and table 1*a*). Temperature remained a significant predictor of prevalence when we allowed for an effect of climatic zone, by estimating a typical prevalence level for each of the five higher-level Köppen climates (‘K5’ in table 1*a*), and when we allowed for systematic differences between all finer-grained Köppen climates (‘K31’ in table 1*a*). Furthermore, the effects of temperature increased when we removed the subset of populations with imprecisely specified locations, where the temperature estimates are least certain (see electronic supplementary material, appendix and table S4a). The improvement in fit due to the Köppen categories suggests that elements of climate, other than temperature, might predict prevalence. This was confirmed by a permutation test, which showed that much smaller improvements were seen in equally large models, but with the Köppen climate labels permuted randomly among the sampled populations (permutation *p*-value <10^−4^; see electronic supplementary material, appendix S1).

**Table 1.** The effects of mean temperature and climatic zone on symbiont prevalence. KC, climatic zones included in the model as categorical predictors, either K5 (five higher-level Köppen classifications) or K31 (up to 31 finer-grained climates); *n*, the total number of parameters in the model fit; pseu-*r*^2^, McFadden's pseudo *r*^2^ [22]; AIC, Akaike information criterion [23], with the preferred model shown in italics; temp: the best-fit slope and *p*-value associated with the mean temperature, when this was included in the model. *p < 0.005*.* The number of arthropod species in each subset of the data is given as a minimum (counting named species only), and a maximum (including each partially identified taxon as a unique species).

dataset:		KC	*n*	AIC	pseu-*r*^2^	temp: slope (*p*-value)	
(*a*) *Cardinium* Mandibulata		—	2	1436.37			
no. populations	1374	—	3	1385.92	0.037	0.128	(7.04 × 10^−13^*)
no. individuals (infected)	11 755 (2070)	K5	6	1434.74	0.007		
no. species min.–max.	623–814	K5	7	1359.13	0.061	0.218	(2.34 × 10^−17^*)
		K31	20	1371.96	0.070		
		K31	21	*1339.39*	0.094	0.198	(3.26 × 10^−9^*)
(*b*) *Wolbachia* Mandibulata		—	2	22 033.92			
no. populations	7986	—	3	22 023.29	0.001	−0.011	(0.000376*)
no. individuals (infected)	102 267 (47 734)	K5	6	21 971.81	0.003		
no. species min.–max.	2451–3686	K5	7	21 953.93	0.004	0.024	(7.79 × 10^−6^*)
		K31	27	*21 741.97*	0.016		
		K31	28	21 743.68	0.016	0.004	(0.590)
(*c*) *Cardinium* Chelicerata		—	2	979.57			
no. populations	345	—	3	*978.14*	0.004	−0.038	(0.064)
no. individuals (infected)	4718 (1212)	K5	6	981.93	0.006		
no. species min.–max.	138–157	K5	7	980.95	0.009	−0.042	(0.083)
		K31	21	980.75	0.038		
		K31	22	982.63	0.038	−0.012	(0.720)
(*d*) *Wolbachia* Chelicerata		—	2	2005.79			
no. populations	638	—	3	2007.68	0.000	−0.005	(0.746)
no. individuals (infected)	8413 (2147)	K5	6	1996.68	0.009		
no. species min.–max.	323–350	K5	7	1998.65	0.009	0.003	(0.843)
		K31	20	1949.50	0.046		
		K31	21	*1949.49*	0.047	0.035	(0.147)

A major caveat to these results is the highly unrepresentative taxonomy in our database (see electronic supplementary material, figure S1 and table S2). For example, over half of the individuals sampled (6127/11 755), and three-quarters of those infected (1596/2070) are Hemiptera (true bugs). Nevertheless, the effect of temperature remained when we removed all Hemiptera from the dataset, and when we considered Hemiptera alone (electronic supplementary material, table S2). Furthermore, the same trend (albeit non-significant) is evident in the two best-sampled hemipteran groups, namely Sternorrhynca (aphids, whiteflies and relatives) and Fulgoromorpha (planthoppers). Together, then, our results suggest that both temperature, and other features of climate have predictable effects on levels of *Cardinium* infection in the Mandibulata.

For *Wolbachia* in Mandibulata (figure 1*g*,*h* and table 1*b*), the results are quite different. Here, there is a significant tendency for colder climates to harbour higher-prevalence infections (table 1*b*). However, this effect is very small (an increase in temperature from 0 to 10°C only decreases expected mean prevalence from 42 to 40%), and not robust to removing populations with imprecisely reported locations (electronic supplementary material, table S4). Nevertheless, similar effects appear in multiple taxonomic groups. As shown in electronic supplementary material, table S2 and figure S4, a negative effect of temperature is found in 4/6 of highly sampled insect orders: Coleoptera, Hymenoptera, Hemiptera and Orthoptera, and in pooled data from the remaining, sparsely sampled groups. The same trend was also seen in mosquitoes (Diptera: Culicidae), and in the remainder of the Diptera, though not in the well-sampled Lepidoptera.

**Figure 2.**
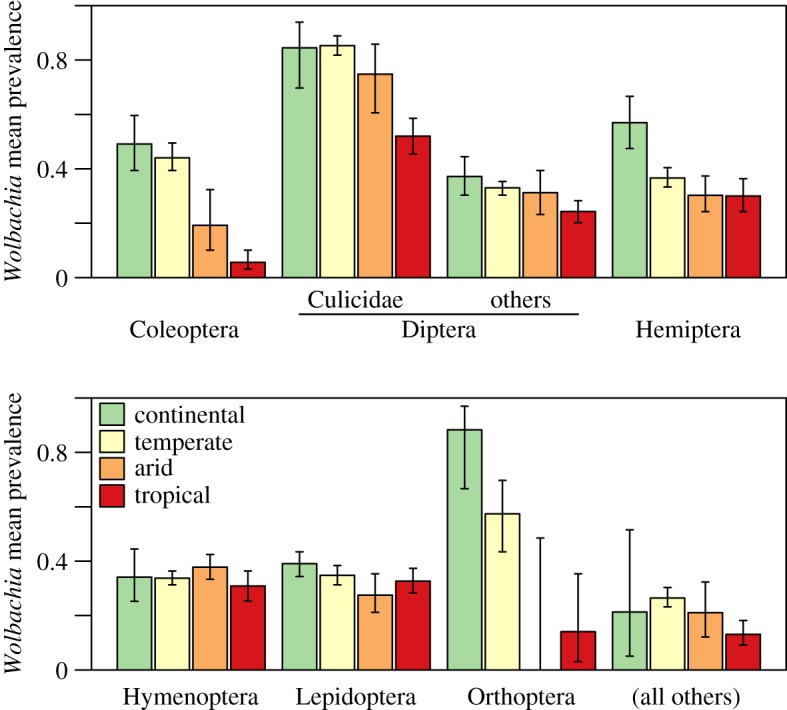
Estimated mean prevalence for *Wolbachia* infection in mosquitoes (Culicidae: Diptera), the best-sampled insect orders, and the remainder of the mandibulate arthropods. In each case, separate estimates are shown for populations from the four best-sampled climatic zones, according to the Köppen system. The main database also contains samples from polar climates, but only a few for each taxonomic group. Confidence intervals correspond to two units of log-likelihood. (Online version in colour.)

As shown in figure 2, this effect is driven by differences between the major climatic zones, with several groups showing less infection in tropical regions, and more infection in cold, continental regions (see also electronic supplementary material, figure S9). If we model this effect, assigning a typical prevalence level to each of the five climates (‘K5’ in table 1*b*), then the effect of temperature reverses sign, and it becomes a significantly positive predictor of prevalence. Finer-grained analyses show that this is driven by a strong effect of temperature within the best-sampled ‘temperate’ zones, with no consistent pattern elsewhere (electronic supplementary material, tables S3 and S4, and figure S5). The effect also disappears if we allow for systematic differences between the finer-grained Köppen climates (‘K31’ in table 1*b*); in this case, model fit improves substantially, and more so than when climatic labels are randomly permuted (permutation *p* < 10^−4^), but including temperature as an explanatory variable adds little predictive power, and fit is very similar for the two largest models.

Chelicerates are much less well-sampled than insects, but this host group showed little evidence of a consistent effect of temperature (figure 1*d*,*e*,*i*,*j*; table 1*c*,*d*). Indeed, for *Wolbachia* there is a notable lack of variation in the mean prevalence estimates. We find meaningful variation between typical prevalences in the finer-grained climatic zones (permutation *p* < 10^−4^), but the best-fit slope for temperature is close to zero (table 1*d*; electronic supplementary material, table S4d), and confidence intervals overlap for most pairs of higher-level climates (see electronic supplementary material, figure S6). This lack of a clear trend holds for the well-sampled Acari (mites, ticks and relatives), which includes many disease vectors, and for the remainder of the chelicerate data (electronic supplementary material, table S2).

## 3. Discussion

Climate and temperature might be associated with symbioses in multiple ways, including direct effects of temperature on the symbiont [7–9] or effects on host density or diversity that might affect symbiont transmission [10,24,25]. A step towards understanding these processes is to determine the patterns of variation that appear in nature. To this end, we have compiled a global database of arthropod populations screened for the *Cardinium* and *Wolbachia*, and tested for an effect of climate, and long-term environmental temperature, on infection prevalence. Our data were extensive, but haphazardly sampled, and so we have focused on patterns we found consistently in different subsets of the data.

A clear and consistent positive relationship between temperature and prevalence was found for *Cardinium* infection in Mandibulata hosts, particularly in insects. This suggests that the pattern reported for *Culicoides* midges from Israel [11] obtains much more generally. While the overall incidence of *Cardinium* in insects is relatively low [3], infection is common in Hemiptera [15], including the rice pest *Sogatella furcifera*, where *Cardinium* appears to increase host fitness [26]. Our results may, therefore, be of relevance to pest control efforts.

For infection with *Wolbachia*—where incidence levels are generally higher—we observed more complex patterns. In chelicerate hosts, no consistent trends were found, and infection levels are surprisingly constant across very different geographical regions, and host groups. In insects, by contrast, we found a tendency for infection to increase with temperature, but only within temperate climates, and tropical climates tended to have lower infection levels overall. This last finding contrasts with previous studies of *Wolbachia* in single species or families of Diptera [13,14], but we observed the trend in multiple host groups, including mosquitoes, the major dipteran disease vectors.

The results for *Wolbachia* have a special interest, because of efforts to use this symbiont as a biocontrol agent, particularly for mosquito-borne human pathogens. For example, in *Aedes aegypti*, *Wolbachia* infection inhibits the replication of dengue, chikungunya and Zika viruses, as well as malarial parasites [5,6,27]. The use of *Wolbachia* for biocontrol has focused on tropical regions (in Australia, Brazil, Indonesia and Vietnam), where the problems of mosquito-borne disease are most acute. But the trends we find within temperate zones suggest that changes in temperature can have consistent effects on symbioses in regions where, because of climate change, such diseases are re-emerging [27,28]. Experimental studies have shown that increases in temperature can perturb mosquito–*Wolbachia* symbioses in the laboratory [29,30], and our results suggest these laboratory results could have a strong ecological relevance.

## 4. Methods

We corrected and extended an existing database of arthropod screens [3], adding data on sampling location. Full details are given in electronic supplementary material, Appendix.

The main statistical analyses used standard beta-binomial modelling [3,31]. Here, the number of infected individuals in a given population sample was treated as a binomially distributed random variable, parameterized with the true prevalence for that population (i.e. the proportion of the population infected). We then assumed that the true prevalence for each population was drawn from a beta-distribution, parameterized with an overall mean prevalence, and a correlation parameter, describing how the variation in infection is distributed within versus between populations [3]. To test for effects of climatic zone and temperature, the logit transformed mean prevalence was constrained to the linear model. For example, we used

$$E\left(\log\left(\frac{\mu_{i}}{1-\mu_{i}}\right)|t_{i},K_{i}\right)=\beta_{0}+\beta_{K_{i}}+\beta_{t}t_{i},$$

where *μ_i_*, is the mean prevalence of population *i*, *t_i_* is the temperature estimate for its sampling location, and *K_i_* is a categorical variable representing its Köppen climate. We held the correlation parameter of the beta-distribution constant across all populations, although relaxing this assumption had little qualitative effect on results (electronic supplementary material, table S2 and figures S8–S11). This agrees with claims that beta-binomial models yield robust estimates of the mean prevalence, even if the shape of the distribution is mis-specified [3]. All models were fitted using the *vglm* function of the R package VGAM v.1.0-3 [32]. Best-fit slopes for temperature, *β_t_*, are reported in table 1 and the best-fit coefficients for the climatic zones, $\beta_{K_{i}}$, in electronic supplementary material, figures S8–S11. Symbiont infection is a highly dynamic process, and so no set of predictors is likely to explain much of the variation [3]. Nevertheless, we also calculated a pseudo-*r*^2^ $(1-\ln\hat{L}/\ln{\hat{L}}_{\mathrm{null}},\text{ where }\hat{L}\text{ is the}$ estimated log-likelihood), [22], and the Akaike information criterion $\left(-2\ln\hat{L}+2n\right)$, which is lower for preferred models [23]. Our major aim was to test for an effect of temperature, and for this purpose, we used two-tailed *z*-tests, defining significance as *p* < 0.005 [33]. For the permutation tests, we recalculated the maximized log-likelihood after randomly permutating the Köppen classifications among populations (following permutation procedures described in [3]). The *p*-value is the proportion of 10 000 random permutations where the maximized log-likelihood is at least as high as with the true classifications. Low *p-*values suggest that the climatic zones are adding predictive power, beyond what would be expected from adding additional parameters to the model.

For the illustrative plots (figure 1*c*,*e*,*h*,*j*; electronic supplementary material, figure S4), we binned populations by mean temperature, centring the bins on the mean temperatures recorded in our dataset for the five higher-level Köppen climates (electronic supplementary material, figure S3). A distinct beta-binomial model was then fitted within each bin, with confidence intervals defined as mean prevalence values that reduce the maximized log-likelihood by two units [34]. The same approach was taken for estimating prevalence in each higher-level climatic zone (see figure 2; electronic supplementary material, figure S6).

## Supplementary Material

Supplementary information

## Supplementary Material

Table S1

## Supplementary Material

Table S2

## Supplementary Material

Table S3

## Supplementary Material

Model code

## Data accessibility

This article has no additional data.
