# Supplementary material for: Wolbachia, Cardinium and climate: an analysis of global data
Source: Biol Lett. 2019 Aug 21;15(8):20190273. doi: 10.1098/rsbl.2019.0273 (PMC6731486; doi:10.1098/rsbl.2019.0273)
Supplement: Supplementary information [file rsbl20190273supp1.pdf]

Supplementary Information

Appendix 1:
In this Appendix we detail the sampling strategy for the database of terrestrial arthropod screens. Reference numbers refer to references in the main text.

Our starting point was the collation of PCR screens of terrestrial arthropods, for *Cardinium, Rickettsia* and *Wolbachia* from 364 publications prior to 2014, published by Weinert et al (3). From this database, we retained only screens for *Wolbachia* and *Cardinium*, from wild populations. Weinert et al. (3) removed screens from areas where host populations were known *a priori* to harbour infection. Given our different goals, we retained these screens, but removed studies where arthropod individuals were pooled before screening. We re-checked entries for all studies, and reentered data in accordance with the goals of the present study (for example, in several instances, multiple geographically proximate populations that were treated as a single large sample in the previous study, were separated here). This led to several changes in previous entries (~0.09% of the total). We then searched the literature for more recent studies, stopping at March 2018. This yielded data from an additional 125 published papers, increasing the number of *Wolbachia* screens by about a third. To associate each record with climate data, we first obtained the decimal latitude and longitude of each sampling location, either from the source publications, or by contacting the authors (see acknowledgements), or by finding named locations on Google Maps (2017). Where location information was very coarse-grained (e.g. "Texas" or "Scotland") we used two strategies to calculate approximate coordinates. If other studies had sampled from the same location, we used the median coordinates of the sampled data for our unspecified location. If no other studies sampled from a given location, we used coordinates for the centroid of that location. Together, these approximate methods were used for ~15.6% of records, as noted in the database. Our results are robust to removing this subset of the data as shown in Supplementary Table S4.

We then used the latitude and longitude information to obtain climatic data. First, we obtained mean temperature estimates over a 2.5-minute grid (~4.5km at the equator) from the WorldClim database (16). We associated each of our sampling locations with its closest grid entry, by Euclidean distance. We also excluded samples from some island or coastal regions, where WorldClim lacks data. Each of our temperature estimates is the mean of weekly maximum and minimum temperatures, averaged over the years 1970-2000. Where sampling dates of the arthropod populations were reported, these were also recorded in our database. But despite the volume of data, there were insufficient records to test directly for temporal change. For similar reasons, we were unable to include other potentially interesting predictors, such as *Wolbachia* supergroups or co-infection with both *Cardinium* and *Wolbachia* in our models. Any discrepancy between sampling dates and temperature estimates should add noise, but not bias to our results. Estimates of temperature variability, and of the maximum and minimum temperatures during 1970-2000, correlate very strongly with the mean temperatures (Supplementary Figure S7), increasing our confidence that our chosen predictor is ecologically meaningful. For the same reason, we used only the mean temperature in our analyses, and did not add additional, highly correlated predictors. We also classified each sampling location according to the Köppen system, again using Euclidean distance, to associate each sampled point with a grid reference (17, data downloaded from 18). These classifications correlate with the mean temperature estimates, and this increases the stringency of our tests, but as shown in Supplementary Figure S3, most climate types

include a wide range of temperatures, and there is overlap between the temperature ranges of different climatic zones.

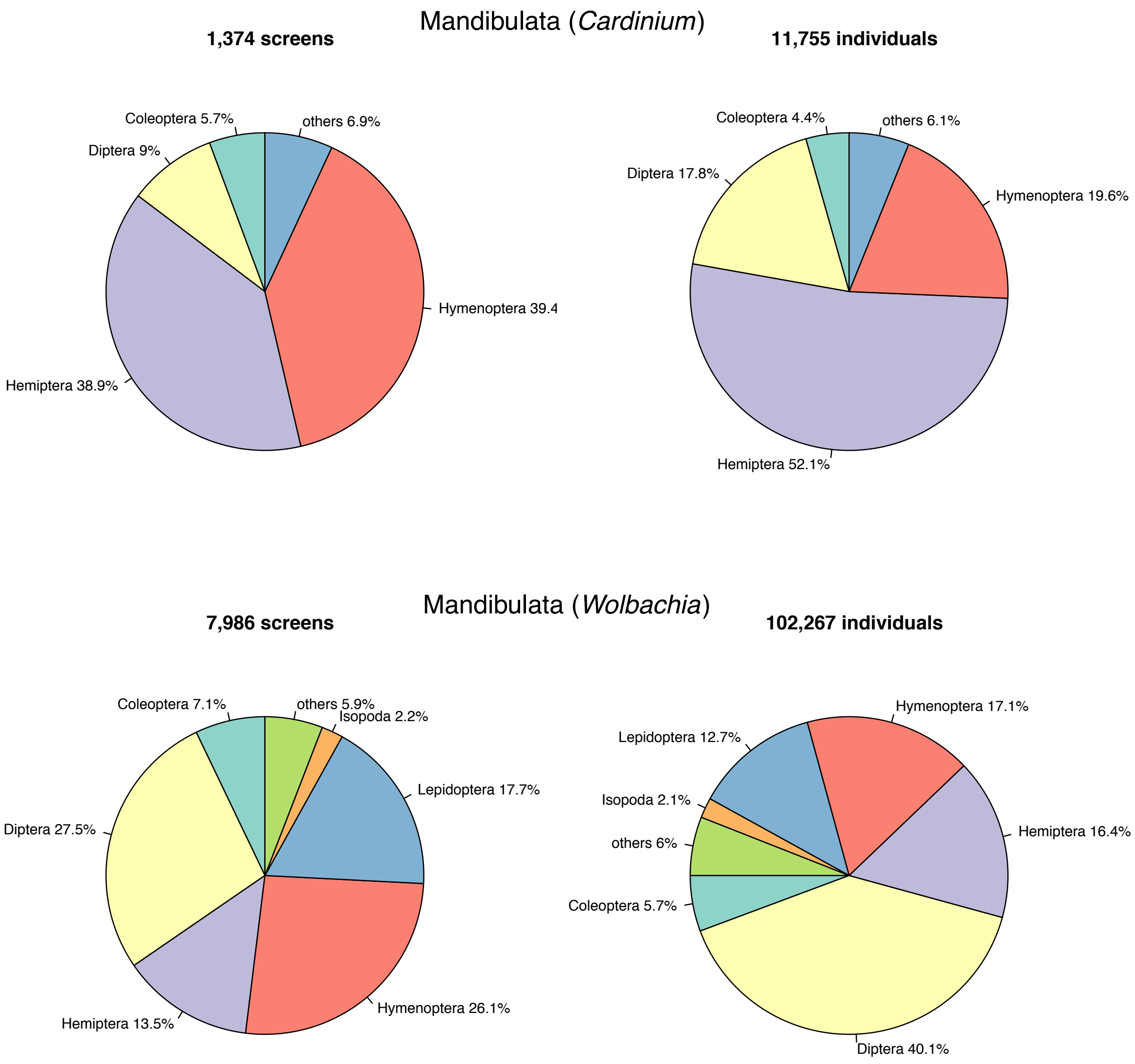

Figure S1:
Taxonomic breakdown of the screen database for arthropod groups classified as Mandibulata, i.e., Hexapoda (including insects), Crustacea and Myriapoda. Plots are shown for the number of screens (i.e., the number of populations sampled), and the total number of individuals screened, excluding pooled screens and samples for which temperature and climate data were not available. Orders are indicated if they comprise >2% of the database, on either measure. The full database is available as Supplementary Table S1.

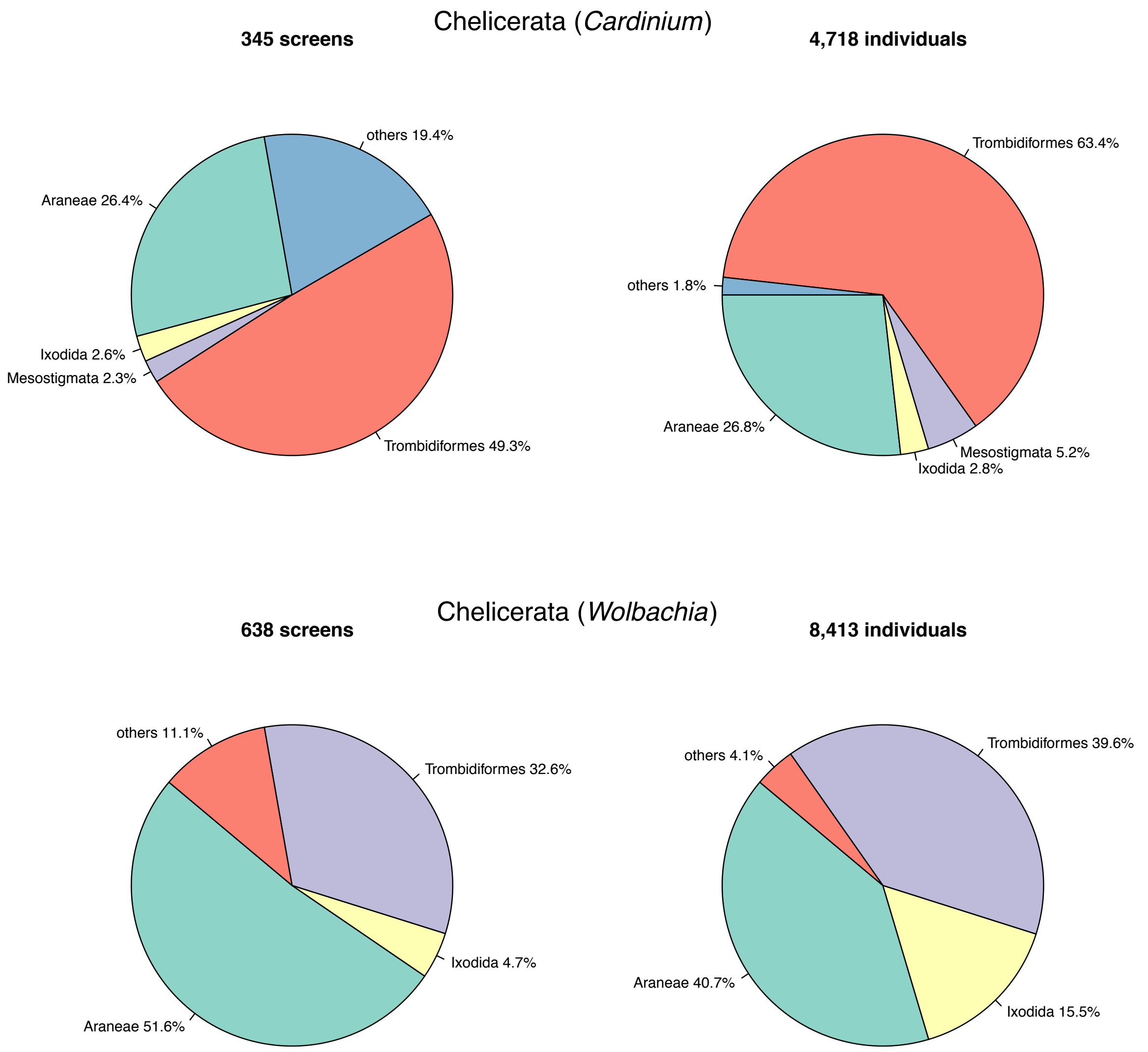

Figure S2:
Taxonomic breakdown of the screen database for arthropod groups classified as Chelicerata, i.e., Acari (ticks and mites), Araneae (spiders), and relatives. All other details match Figure S1.

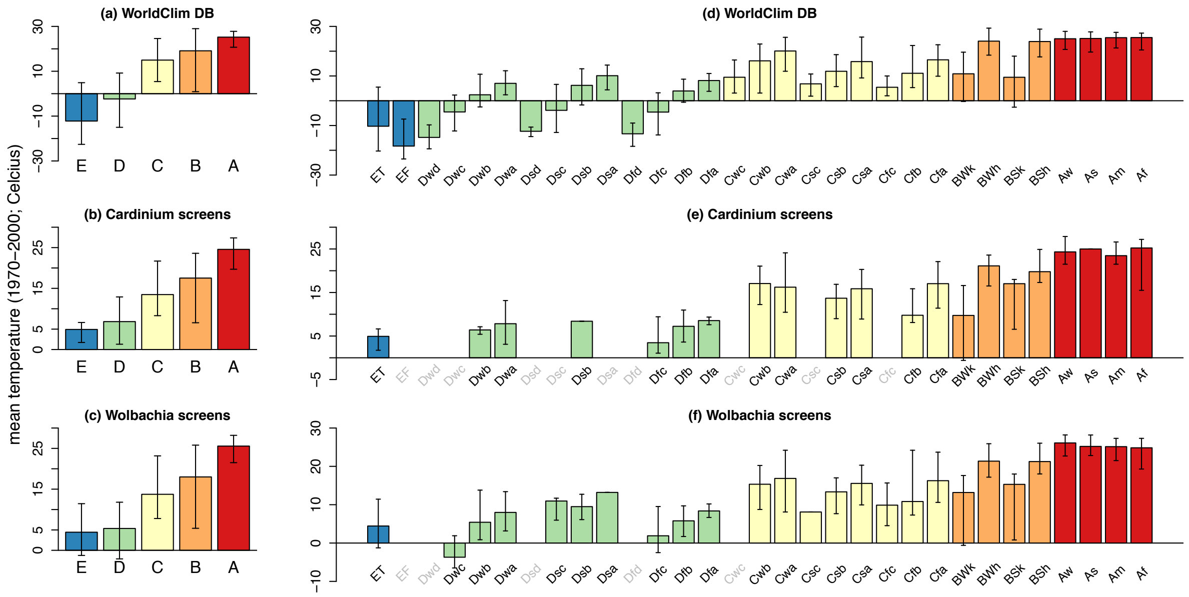

Figure S3:
The range of mean monthly temperatures for the period 1970-2010 associated with each of the Köppen climate classifications. These classifications are fit as a categorical predictor, along with mean temperature itself, in our analyses of symbiont prevalence (see Table 1; Supplementary Table S2). Bars show means, and confidence intervals show 95% quantiles. The upper row (panels (a) and (d)) shows all records in the WorldClim 2 database (Fick and Hijmans 2017). The middle row (panels (b) and (e)) shows the equivalent results for locations in our database of *Cardinium* screens, and the lower row (panels (c) and (f)) shows results for our database of *Wolbachia* screens. The left-hand column (panels (a)-(c)), shows results for the five higher-level climate classifications, namely A (tropical), B (arid), C (temperate), D (continental), and E (polar). The right-hand column (panels (d)-(f)): shows results for the 31 finer-grained climates, namely: Af (tropical rainforest), Am (tropical monsoon), Aw (tropical savanna, wet), As (tropical savanna, dry); BWh (hot desert), BWk (cold desert), BSh (hot steppe), BSk (cold steppe), Csa (hot-summer Mediterranean), Csb (warm-summer Mediterranean), Csc (cool-summer Mediterranean), Cwa (Monsoon-influenced humid subtropical), Cwb (subtropical highland or temperate oceanic climate with dry winters), Cwc (cold subtropical highland climate or subpolar oceanic climate with dry winters), Cfa (humid sub-tropical), Cfb (temperate oceanic), Cfc (subpolar oceanic); Dfa (hot-summer humid continental), Dfb (warm-summer humid continental), Dfc (subarctic), Dfd (extremely cold subarctic), Dwa (Monsoon-influenced hot-summer humid continental), Dwb (Monsoon-influenced warm-summer humid continental climate), Dwc (Monsoon-influenced subarctic climate), Dwd (Monsoon-influenced extremely cold subarctic climate); Dsa (hot, dry-summer continental), Dsb (warm, dry-summer continental), Dsc (sub-arctic, dry-summer continental), Dsd (extremely cold sub-arctic, dry-summer continental); ET (polar tundra), EF (eternal winter/ice cap). Comparing the rows shows that the some of the finer-grained climates were missing from our population sample (indicated by the gray labels), while others were represented by a biased sample with respect to temperature. For example, there were no population samples from EF (eternal winter/ice cap), while samples from ET (polar tundra) come from the warmer parts of this climatic zone.

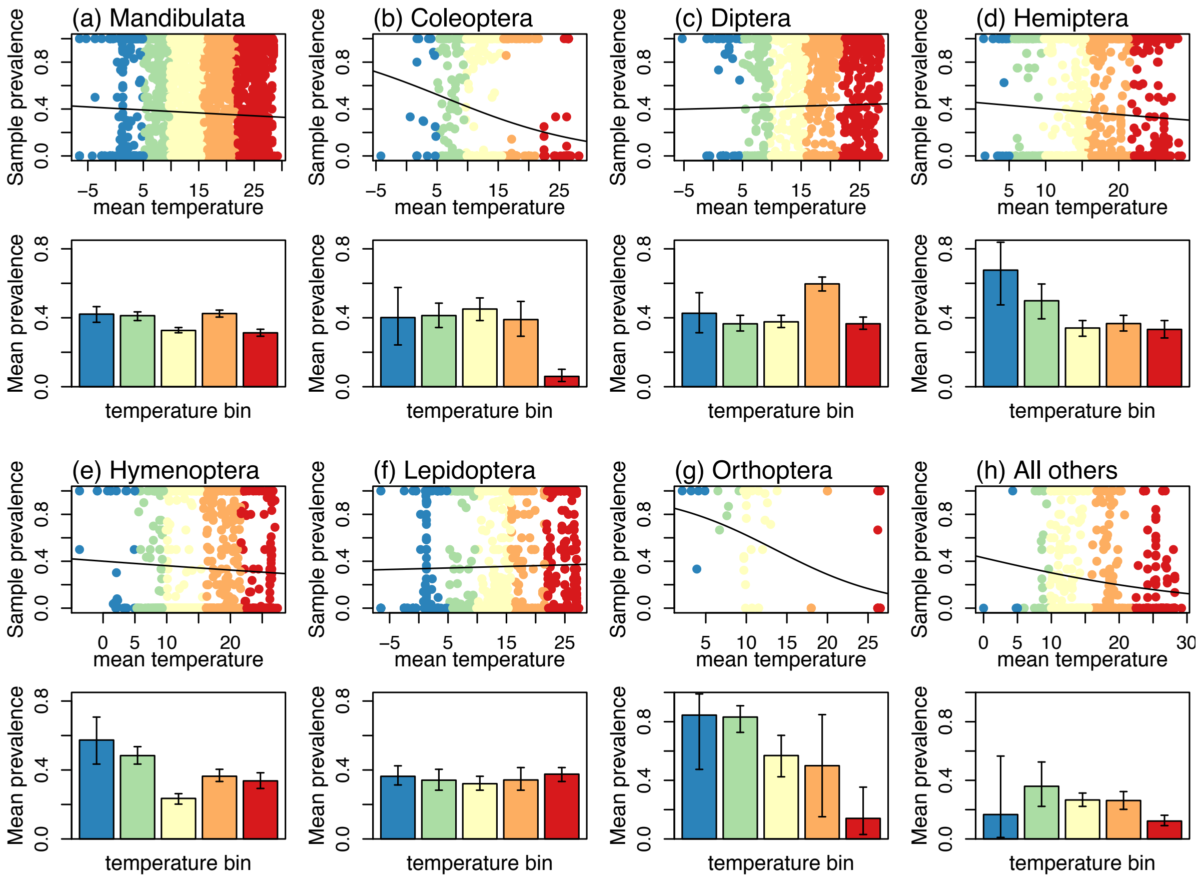

Figure S4:
The effects of temperature on *Wolbachia* prevalence in the major insect orders (panels (b)-(g)), and in the remainder of the sampled Mandibulata (panel (h)). The slopes correspond to results for three-parameter models given in Supplementary Table S2. All other details match Figure 2b-c, which is replicated for ease of comparison in panel (a).

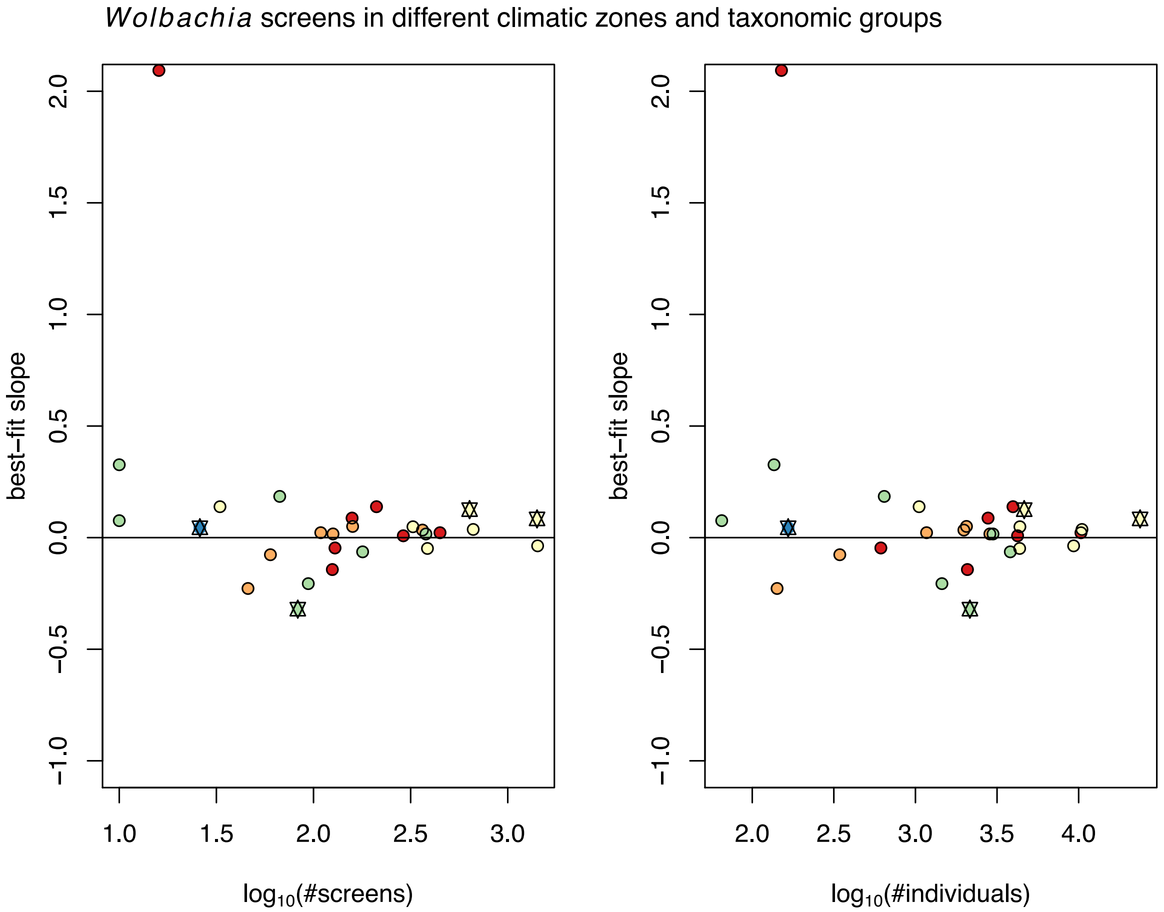

Figure S5:
Best-fit slopes for the regression of mean *Wolbachia* prevalence onto temperature. Each point represents samples from a particular group of mandibulate hosts (one of the major insect orders, or the remainder of the data), and a single higher-level Köppen zone (polar: blue; continental: green, temperate: yellow, arid: orange; and tropical: red). Regressions that were individually significant are indicated as asterisks, and a full description is found in Supplementary Table S3. As expected, data show that the estimates of the slopes become more erratic as sample size decreases, but there is little evidence for a consistent trend towards negative or positive slopes. The apparent trend for *Wolbachia* infection to increase with temperature is driven by the well-sampled Diptera and Lepidoptera in temperate zones, indicated by the yellow asterisks towards the right-hand side of the plots.

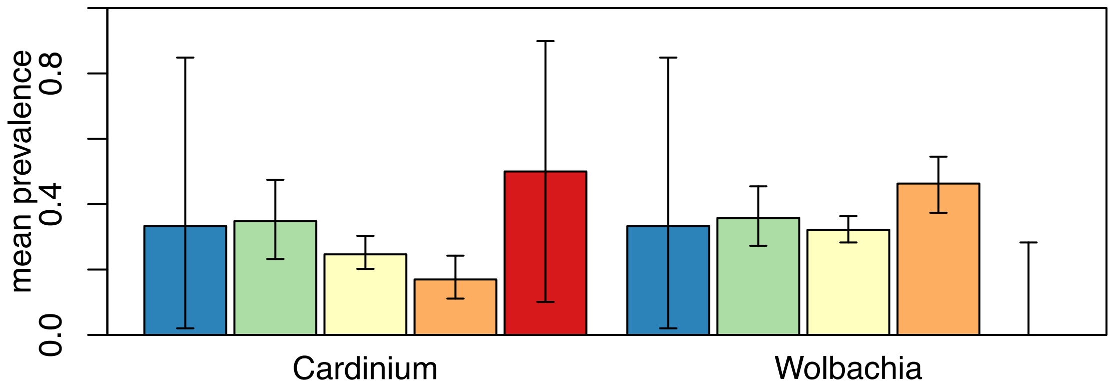

Figure S6:
Best-fit mean prevalence estimates in Chelicerata hosts, for the symbionts *Cardinium* (left-hand bars) and *Wolbachia* (right-hand bars). In each case, estimates are shown for the five higher-level Köppen climates: from left-to-right, these are polar (blue), continental (green), temperate (yellow), arid (orange) and tropical (red).

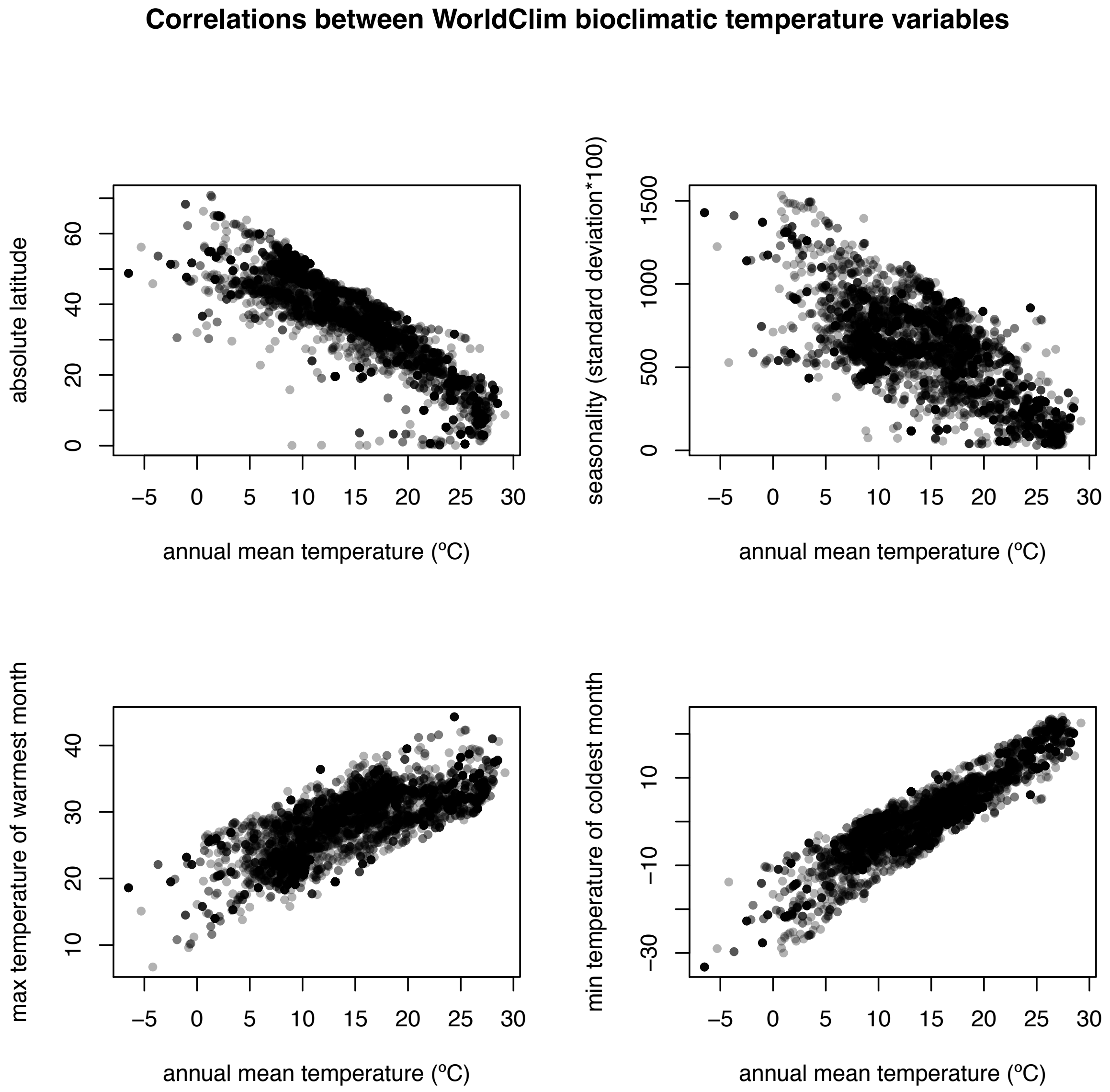

Figure S7:
Estimates of mean temperature show strong correlations with other potential predictors of endosymbiont prevalence. Results are shown for (a) absolute latitude, (b) "seasonality" (defined as the standard deviation in the recorded temperatures over the period 1970-2000), (c) the maximum recorded temperature over this period, and (d) the minimum recorded temperature. All data come from the WorldClim database (Fick and Hijmans 2017).

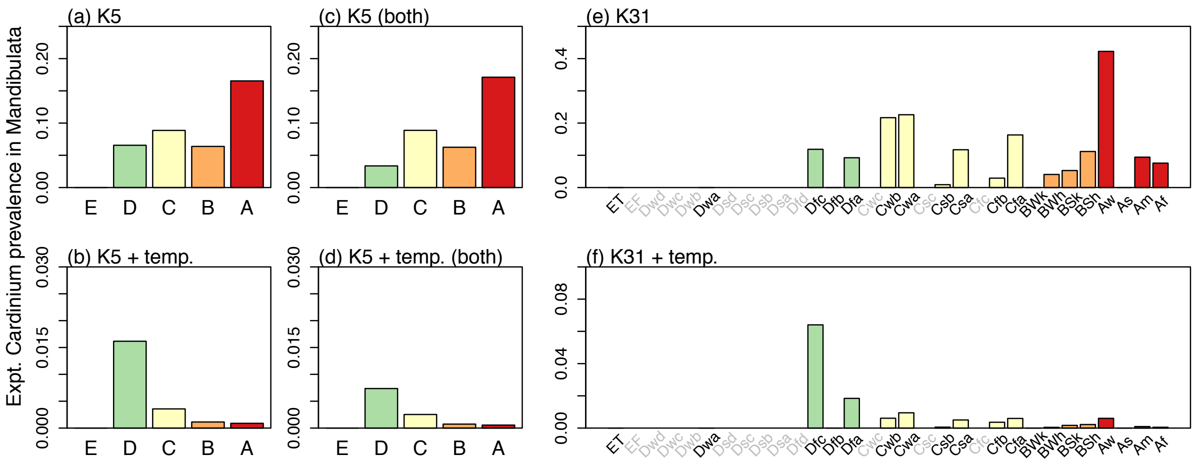

Figure S8:
Best-fit mean prevalence values for *Cardinium* prevalence in Mandibulata hosts, in each of the Köppen climate zones. The upper row of panels ((a), (c) and (e)) shows estimates when only the climatic zones were included in the model, while the lower row (panels (b), (d) and (f)), show estimates after the effects of mean temperature have been removed. The strong difference between these rows suggests that temperature is a strong predictor of mean prevalence in these data. Results are compared for the five higher-level classifications (panels (a)-(d)), and for the 31 finer-grained classifications (panels (e)-(f)), not all of which were represented in our sample of screens (shown by the gray labels). Results are also compared when the model was fit only to mean prevalence (panels (a)-(b)), and when it was also fit the correlation parameter, allowing the shape of the distribution of prevalences to vary between climate zones (panels (c)-(d)). The definitions of all climatic zones, and the range of temperatures that they encompass, are shown in Supplementary Figure S3.

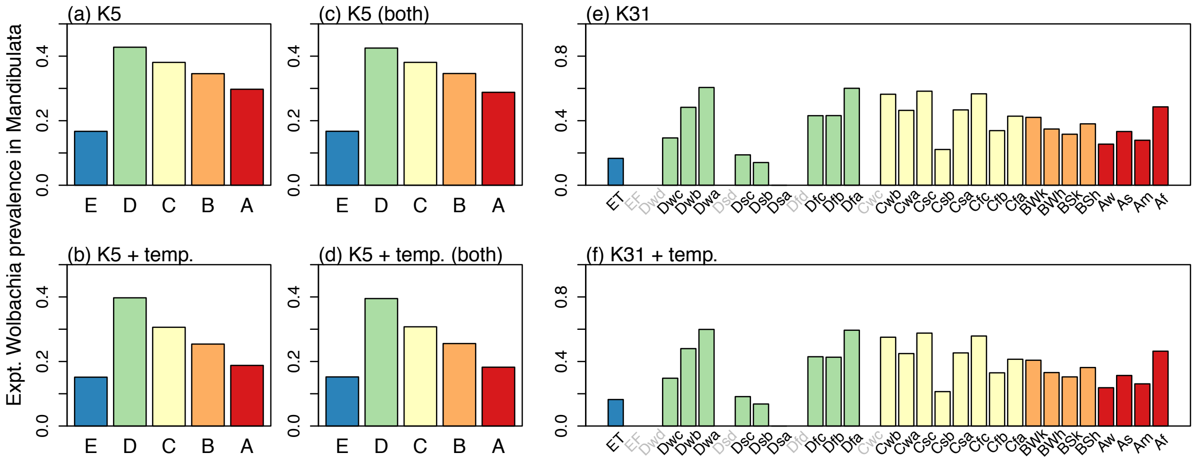

Figure S9

Best-fit mean prevalence values for *Wolbachia* prevalence in Mandibulata hosts, in each of the Köppen climate zones. The broad similarity between the upper and lower row of panels suggests that mean temperature is not a strong predictor of prevalence for these data. All other details match Supplementary Figure S8.

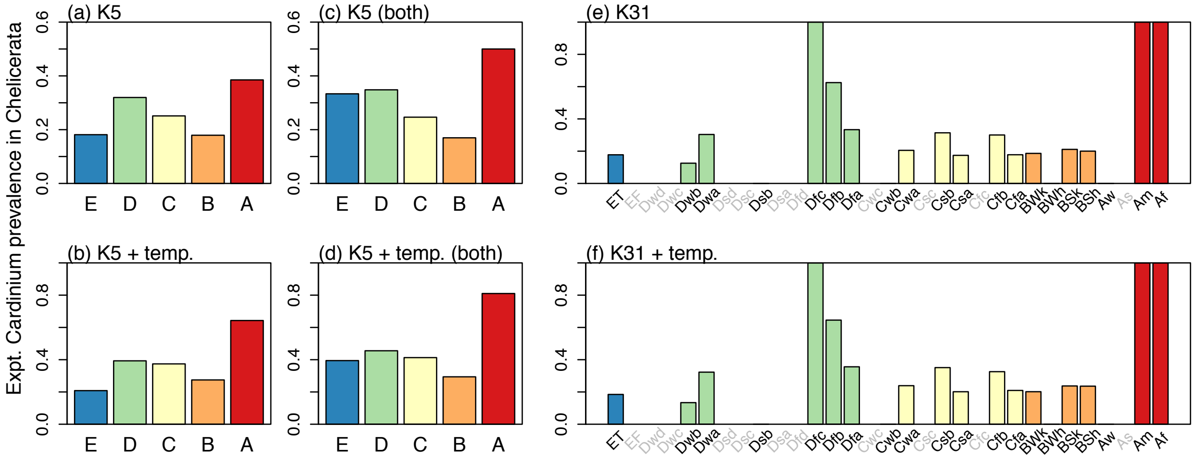

Figure S10:

Best-fit mean prevalence values for *Cardinium* prevalence in Chelicerata hosts, in each of the Köppen climate zones. The broad similarity between the upper and lower row of panels suggests that mean temperature is not a strong predictor of prevalence for these data. All other details match Supplementary Figure S8.

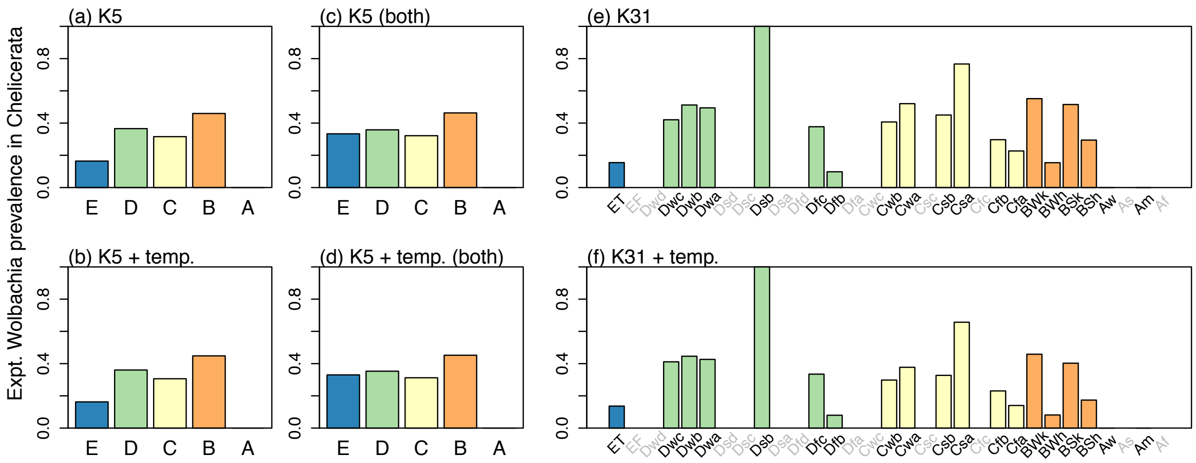

Figure S11:
Best-fit mean prevalence values for *Wolbachia* prevalence in Chelicerata hosts, in each of the Köppen climate zones. The broad similarity between the upper and lower row of panels suggests that mean temperature is not a strong predictor of prevalence for these data. All other details match Supplementary Figure S8.

Description of Supplementary Tables:
Supplementary Table S1: The database of screens of wild arthropod populations either *Cardinium* or *Wolbachia*.

Supplementary Table S2: Results of Beta-binomial model fitting to symbiont screen data.

Supplementary Table S3: Tests for an effect of mean temperature of *Wolbachia* prevalence in Mandibulata hosts, applied separately to populations from each of the five higher-level Köppen climates.

Supplementary Table S4: The effects of mean temperature and climatic zone on symbiont prevalence, excluding imprecisely reported locations (as described in Appendix 1).

**Data set:**		KC	*np*	AIC	pseu-r^2	temp: slope	(*p* val)
**(a) *Cardinium* Mandibulata**		-	2	1136.43			
# Populations:	921	-	3	1028.50	0.097	0.237	(5.47×10^{-22}*)
# Individuals (infected):	10,451 (1,985)	K5	5	1111.23	0.028		
		K5	6	1002.71	0.125	0.306	(1.34×10^{-22}*)
		K31	17	1014.18	0.134		
		K31	18	**983.31**	0.163	0.232	(5.26×10^{-9}*)
**(b) *Wolbachia* Mandibulata**		-	2	19107.00			
# Populations:	6,749	-	3	19109.00	0.000	0.000	0.987
# Individuals (infected):	89,023 (43,890)	K5	6	19085.01	0.002		
		K5	7	19056.85	0.003	0.031	(3.83×10^{-8}*)
		K31	26	18865.14	0.015		
		K31	27	18863.52	0.015	0.014	0.055
**(c) *Cardinium* Chelicerata**		-	2	929.29			
# Populations:	327	-	3	**924.82**	0.007	-0.054	0.0119
# Individuals (infected):	4,067 (1,119)	K5	6	931.88	0.006		
		K5	7	928.57	0.012	-0.058	0.0223
		K31	21	931.60	0.039		
		K31	22	932.29	0.040	-0.041	0.0240
**(d) *Wolbachia* Chelicerata**		-	2	1586.08			
# Populations:	459	-	3	1587.49	0.000	-0.012	0.439
# Individuals (infected):	6,378 (1,875)	K5	6	1582.68	0.007		
		K5	7	1584.63	0.007	0.004	0.825
		K31	20	**1553.69**	0.043		
		K31	21	1553.87	0.044	0.035	0.172

Note: KC: climatic zones included in the model as categorical predictors, either K5 (five higher-level Köppen classifications) or K31 (up to 31 finer-grained climates); *np*: the total number of parameters in the model fit; pseu-r^2: McFadden's pseudo r-squared (30); AIC: Akaike Information Criterion (31), with the preferred model shown in bold; temp: the best-fit slope and p-value associated with the mean temperature, when this was included in the model. * $p < 0.005$. The number of arthropod species in each subset of the data is given as a minimum (counting named species only), and a maximum (including each partially identified taxon as a unique species).
